# Refining the genomic profiles of North African sheep breeds through meta-analysis of worldwide genomic SNP data

**DOI:** 10.3389/fvets.2024.1339321

**Published:** 2024-02-29

**Authors:** Imen Baazaoui, Sonia Bedhiaf-Romdhani, Salvatore Mastrangelo, Johannes A Lenstra, Anne Da Silva, Badr Benjelloun, Elena Ciani

**Affiliations:** ^1^Laboratory of Animal and Fodder Production, National Institute of Agronomic Research of Tunisia, Ariana, Tunisia; ^2^Dipartimento Scienze Agrarie, Alimentari e Forestali, University of Palermo, Palermo, Italy; ^3^Faculty of Veterinary Medicine, Utrecht University, Utrecht, Netherlands; ^4^Faculté des Sciences et Techniques de Limoges, E2LIM, Limoges, France; ^5^National Institute of Agronomic Research (INRA Maroc), Regional Centre of Agronomic Research, Beni Mellal, Morocco; ^6^Dipartamento Bioscienze, Biotecnologie, Biofarmaceutica, University of Bari Aldo Moro, Bari, Italy

**Keywords:** North African sheep, population structure, worldwide SNP data, genetic gradients, signature of selection, local ancestry

## Abstract

**Introduction:**

The development of reproducible tools for the rapid genotyping of thousands of genetic markers (SNPs) has promoted cross border collaboration in the study of sheep genetic diversity on a global scale.

**Methods:**

In this study, we collected a comprehensive dataset of 239 African and Eurasian sheep breeds genotyped at 37,638 filtered SNP markers, with the aim of understanding the genetic structure of 22 North African (NA) sheep breeds within a global context.

**Results and discussion:**

We revealed asubstantial enrichment of the gene pool between the north and south shores of the Mediterranean Sea, which corroborates the importance of the maritime route in the history of livestock. The genetic structure of North African breeds mirrors the differential composition of genetic backgrounds following the breed history. Indeed, Maghrebin sheep stocks constitute a geographically and historically coherent unit with any breed-level genetic distinctness among them due to considerable gene flow. We detected a broad east–west pattern describing the most important trend in NA fat-tailed populations, exhibited by the genetic closeness of Egyptian and Libyan fat-tailed sheep to Middle Eastern breeds rather than Maghrebin ones. A Bayesian F_ST_ scan analysis revealed a set of genes with potentially key adaptive roles in lipid metabolism (*BMP2, PDGFD VEGFA, TBX15,* and *WARS2*), coat pigmentation (*SOX10, PICK1, PDGFRA, MC1R,* and *MTIF*) and horn morphology *RXFP2*) in Tunisian sheep. The local ancestry method detected a Merino signature in Tunisian Noire de Thibar sheep near the *SULF1*gene introgressed by Merino’s European breeds. This study will contribute to the general picture of worldwide sheep genetic diversity.

## 1 Introduction

The sheep (*Ovis aries*) was domesticated approximately 11,000 years before present in the Fertile Crescent from Asian Mouflon (*Ovis orientalis*). Thousands of years of artificial selection coupled with human-driven migration and adaptation to diverse environmental conditions resulted in more than 1,000 distinct sheep breeds reared in different parts of the globe, which possess unique genetic profiles adapted to local economic needs (1). The North African (NA) sheep is 7,000 years old and represents a remarkable diversity of sheep populations reared under traditional farming systems over millennia. The history of African sheep indicates that the most ancient sheep migrated from the domestication center into Africa, perhaps in response to droughts and unstable climates (2). Then, the overland dispersal route of sheep was northward to Libya (6500–6,800 BP), southward to the central Nile Valley (6,000 BP), and westward to the central Sahara (6,000 BP), reaching west Africa by 3,700 BP (3). For millennia, Mediterranean maritime trading routes have facilitated the dispersal of sheep along the North African coastline, often accompanied by hybridization between local and exogenous breeds imported by settlers into the area. These movements reshaped the genetic differentiation of local sheep genetic resources (4). Most of the current NA sheep genetic resources constitute a geographically and historically coherent unit well adapted to local conditions. Currently, these breeds have been subject to recent intermixing and genetic homogenization, leading to the creation of crossbred populations (5–7). In the Maghreb region (Tunisia, Algeria, and Morocco), most sheep breeds are coarse-wool thin-tailed sheep, with the exception of fat-tailed Barbarine ecotypes. Some transboundary populations are also found in this region. These consist of Moroccan D’man, which is also present in Tunisia and Algeria, and the Algerian Ouled Djallel, which is kept as well in Morocco and under the name Queue Fine de l’Ouest in Tunisia. However, the African northeast is home to exclusive fat-tailed breeds located in Egypt and Libya. Artificial selection appears to play a minor role in driving the genome evolution of these populations, which were rather endowed with resilience traits shaped by local adaptation to natural environments. Although they may not be commercially valuable, they are endowed with unique abilities to survive in a particular environment or disease (8). The detection of signatures of selection in sheep populations originating from different geographical origins may detect potential candidate genes associated with different ecological adaptations (9). To date, several genome-wide SNP studies identified the genomic basis of adaptation of NA local sheep breeds to semi-arid (7, 10), hot desert conditions (11, 12), tolerance to environmental diseases (13–16), and signatures of domestication (1). The development of affordable and reproducible tools for rapid genotyping of thousands of genetic markers (SNPs) has promoted cross-border collaboration in the study of sheep genetic diversity on a global scale (17). Since then, the scientific community has focused on small-scale genome-wide diversity at a national level, which has limited the scope for continent-wide interpretations of research findings. In order to partially fill this gap, global-scale phylogeographic studies have focused on sheep genetic variation and history on worldwide (18), European (19–21), Sub-Saharan African (22), East Asian (23), West Asian (24) and north-west African (6, 25) geographical scales. North Africa has shown an important genetic diversity of indigenous sheep breeds whose origin is associated with its environmental characteristics and to certain historical events in the region. Until now, there is rare archeological evidence that trace back the complex scenario in reconstructing the history of NA sheep breeds. In this study, we assembled a large worldwide sheep dataset to (i) provide a closer examination of genetic relationships and admixture patterns, among NA breeds from Africa and Eurasia (ii) detect genomic regions/genes under selection, in some Maghrebin sheep, having distinct historical origin and raised in divergent agro-ecological regions. This study contributes another piece to the general picture of worldwide sheep diversity and gives a deeper insight into the genomic profile of local sheep adaptation.

## 2 Materials and methods

### 2.1 Sheep breeds and samples

This study represents a meta-analysis of a worldwide genomic SNP dataset mostly represented by African and Eurasian sheep breeds derived from publicly available datasets. Detailed information about sheep populations and samples is given in Supplementary Table S1. Data was obtained from a total of 234 domestic sheep breeds originating from Africa (*n* = 40 breeds) (1, 5–7, 10, 17, 21, 22, 26–28), Europe (*n* = 148 breeds) (17, 19–21, 29–33) and Asia (*n* = 46 breeds) (17, 27, 34–38). The North African samples were represented by 80 samples originating from 22 breeds from the Maghreb region [Morocco (*n* = 60), Algeria (*n* = 64) and Tunisia (*n* = 59)] and Libya and Egypt (*n* = 44). Five additional wild populations (*n* = 53 individuals) (20) (Argali, Urial, three mouflon populations from Europe, Sardinia and Asia) were used as an out-group for studying the stratification of studied breeds.

### 2.2 Data management

All samples were genotyped using the ovine SNP50K BeadChip with the exception of French breeds (*n* = 23) which were genotyped using Illumina Ovine HD SNP chip. The SNP coordinates were updated to the Ovis_aries_v4.0 version using the information available in the SNPchiMP v3 database (39). The merging of data generated with Illumina Ovine HD SNP chip with ovine SNP50 BeadChip data was based on common SNPs between them. We used PLINK 1.9 (40)^1^ for data merging and quality checks. The combined raw dataset was filtered to exclude duplicate samples and poorly genotyped individuals (<90% of SNPs). SNP markers (i) located on sexual chromosomes (ii) genotyped in <90% of individuals and (iii) with rare alleles (MAF < 0.01) were also excluded. We retained 37,638 SNPs after linkage disequilibrium filtering using the following parameters: 50 SNPs per window, a shift of 5 SNPs between windows, and a variation inflation factor’s threshold of two (corresponding to *r*^2^ > 0.5). To reduce the bias from over-represented breeds, data were restricted to a maximum of 24 animals per breed, selected at random using the –thin-indiv command in PLINK 1.9, resulting in a dataset of 4,082 individuals in 234 domestic and five wild populations. In this study, we created reduced datasets useful for targeted genomic studies.

### 2.3 Analysis of population structure

#### 2.3.1 Genetic structure of North African sheep in the global context

We examined the genetic relationships between 22 breeds that originated in the Maghreb region (Morocco, Algeria, and Tunisia), Libya, and Egypt with 217 breeds from different geographical distributions. The genetic relationships between populations were estimated using a matrix of genome-wide identity-by-state (IBS) genetic distances calculated by PLINK 1.9 and plotted using MDS plots focusing on different geographic cross-border contexts. The allele sharing distance was used to perform a neighbor joining (ASD-NJ) phylogenetic tree using the Splits Tree 4.13.1 software (41) in order to visualize the complex population networks between 80 NA sheep samples and 300 fat-tailed individuals from Africa. The admixture patterns were assessed by the maximum likelihood-based approach implemented in the ADMIXTURE software v1.3.0 (42) by applying the default settings. In addition, unsupervised and supervised hierarchical clustering of individuals, were performed to examine patterns of ancestry and admixture in different datasets.

#### 2.3.2 Genetic structure of North African breeds at macro-geographical scale

To better resolve the genetic relationships between NA breeds, we selected a data subset, taking into account possible connections with breeds/populations presumed to have contributed to shaping the current genetic background of some of them. Thus, we carried out an MDS and unsupervised admixture analysis of 22 north African breeds and four representative breeds from Europe (SWBB, MEAR), sub-Saharan Africa (WAD) and the Middle East (LOAW). The same dataset was used to efficiently capture information on population structure provided by patterns of haplotype similarity. Each individual in a sample is considered in turn as a recipient, whose chromosomes are reconstructed using chunks of DNA donated by the other individuals. The results of this “chromosome painting” can be summarized as a “coancestry matrix,” which directly reveals key information about ancestral relationships among individuals. The dataset was filtered (39,454 SNPs) and phased haplotypes were inferred using the Shapeit v2. software (43). Shared haplotypes were identified using the program Chromopainter v2 and posterior distribution of clusters were visualized via the associated fineStructure v2 tree-building algorithm (44).

### 2.4 Detection of genomic regions under selection

#### 2.4.1 Detection of F_ST_ outlier loci

The *F_ST_* differentiation index is one of the most commonly used metrics in detecting signatures of selection in animals (45). Thus, we used the hierarchical-Bayesian model implemented in BayeScan 2.1 software (46) to detect markers putatively under differential selection pressure, using two sheep breeds from Tunisia (Barbarine and Noire de Thibar), characterized by specific adaptive and morphological traits. This package implements an F_ST_-based hierarchical Bayesian model using a reversible jump MCMC (Monte Carlo Markov Chain). Thus, we used six pairwise comparisons to detect putative loci under selection associated with fat tail deposition (BART vs. thin-tailed breeds) and coat pigmentation (NOTH vs. white coated breeds) (Supplementary Table S2). The contrasted breeds were representatives of different geographical origins from North Africa (QFOT, NOTH, BART, ODJA, and ODMA), West Africa (WAD), southern Europe (BERG), and Asia (TIBT and CHANG). For each cohort, loci that displayed a q value <0.05 were considered as being under selection. In order to avoid the detection of false positive loci and define chromosome regions putatively under differential selection, we identified loci that differed significantly in at least three pair-wise comparisons for each trait. For these loci, we then considered a window of ±200 kbp upstream and downstream of the significant markers (47). Annotated genes within the genomic regions putatively under selection were obtained by querying the Genome Data Viewer for *Ovis aries* (available online)^2^ and their biological functions were investigated with a systematic literature review.

#### 2.4.2 Inference of European local ancestry

Information about patterns of genetic ancestry in admixed livestock populations provides insights into past introgression events and genomic regions under selection. The Tunisian Noire de Thibar (NOTH) sheep was created at the beginning of the 20th century and is considered an ideal model of composite breed shaped by crossbreeding between local (QFOT) and European stocks (MEAR and SWBB) (13). Local Ancestry in Mixed Populations (LAMP) is a program for estimating locus-specific ancestries in 20 NOTH admixed individuals, using allele frequencies of the reference populations (48). We applied the LAMP and LAMP-ANC modes implemented in LAMP and provided the estimated allele frequency files for one local and two European breeds as the purebred ancestral populations using three different scenarios (Table 1). The following default settings were adopted: number of generations since admixture (g) = 32 and recombination rate (r) =10^−8^. The fraction of global admixture (α) was determined, for each scenario, using the ADMIXTURE software (42). We consider the adjacent SNPs with the highest value of MAA at the autosome-wide level to be the significant region under selection. We conducted a comprehensive search in the available literature and public databases to investigate the biological function and the phenotypes that are known to be affected by each annotated gene.

**Table 1:** Tested scenarios of NOTH creation using one local and two European breeds as the purebred ancestral populations.

Approach	SNPs	Ancestry1: QFOT		Ancestry 2: MEAR			Ancestry 3: SWBB		
		*n*	*α*	*n*	*α*	MAA	*n*	*α*	MAA
LAMP	39,254	13	0.90	-	-		23	0.1	0.21–0.43
LAMP	40,574	13	0.83	23	0.17	0.07–0.33	-	-	-
LAMP-ANC^1^	39,370	13	0.81	23	0.17		18	0.02	

^1^LAMP-ANC is a modification of the LAMP mode and shows higher accuracy allowing triple mixing (QFOT x MEAR x SWBB) to be estimated, while LAMP cannot determine frequencies for more than two ancestral populations (QFOT x MEAR/QFOT x SWBB).

*α*: fraction of global admixture.

## 3 Results

### 3.1 Genetic structure of North African sheep populations in a global context

**Figure 1:**
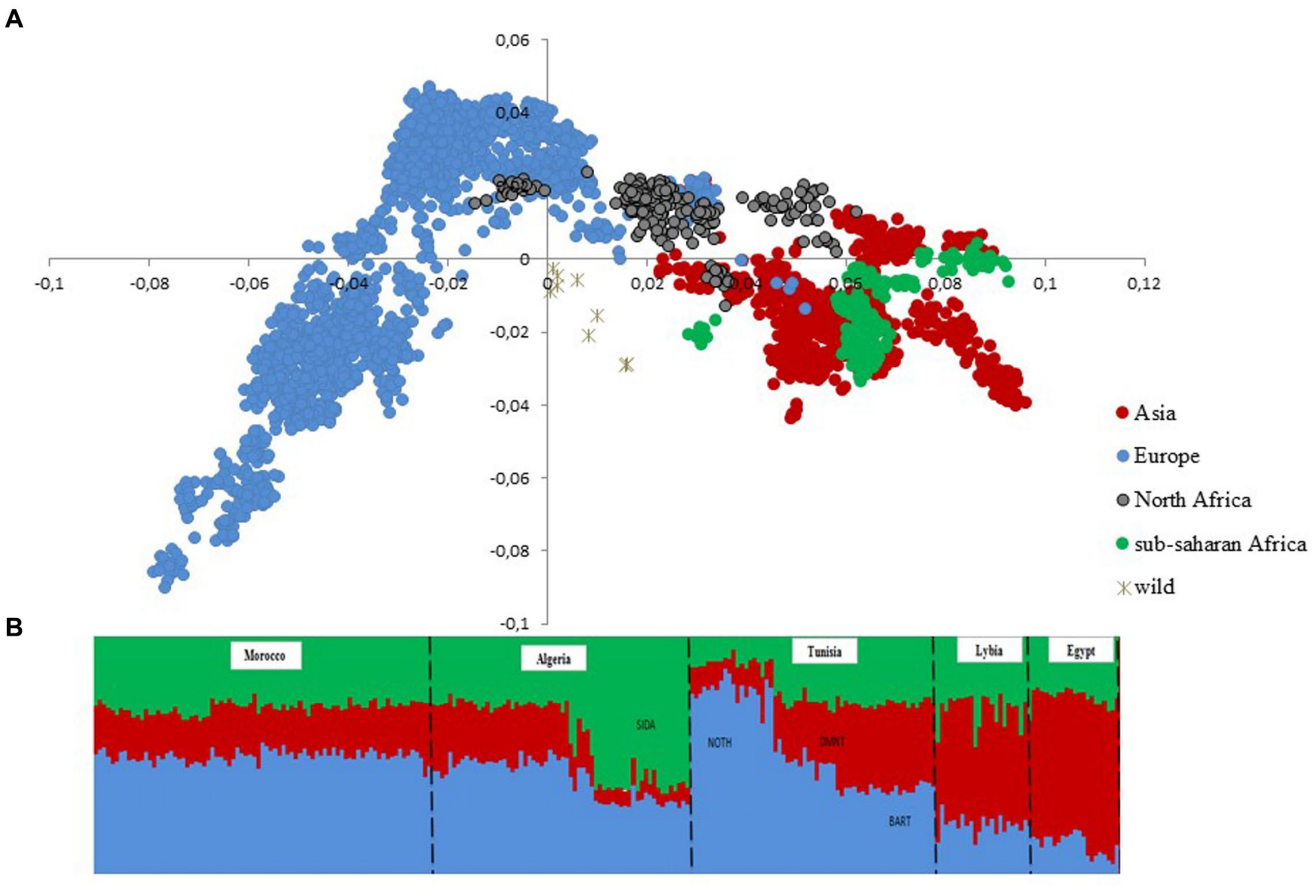
Genetic structure of 22 North African sheep breeds in a worldwide context using 4,083 samples genotyped in 37,638 SNPs. **(A)** Multi-dimensional scaling **(B)** Model-based clustering supervised (*K* = 3) analysis. Only breeds from North Africa are shown with prior information given to breeds originating from sub-Saharan Africa (green), Europe (blue), and Asia (red).

**Figure 2:**
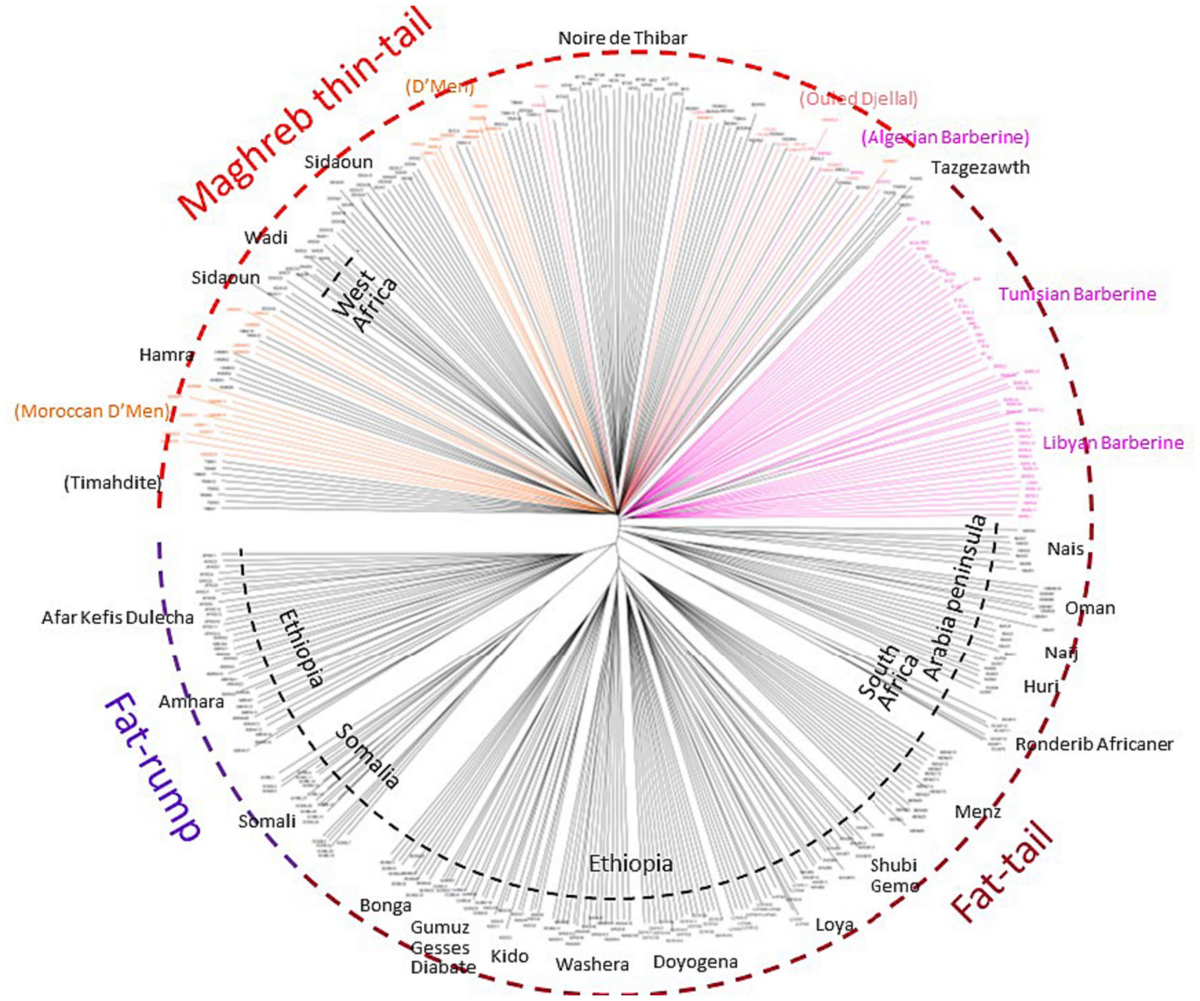
The Neighbor-Joining tree based on the shared-allele genetic distance showing a clustering pattern of 379 African fat-tailed sheep based on 37,778 SNP markers.

After quality control, the merged dataset used in the analyses consisted of 4,082 individuals from 234 domestic sheep and five wild populations (Supplementary Table S1). According to the MDS analysis (Figure 1A), we noticed a clear distinctiveness of domesticated breeds from wild populations (Argali, Urial, and Asiatic Mouflon) occupying the center of the plot. The second axis splits the worldwide breeds into two main groups, where the European sheep breeds are located on the left side of the plot, with the exception of Tunisian NOTH samples. The right-side group consists of Mediterranean samples from North Africa, Greece, Cyprus, Canary Islands, and samples from sub-Saharan African and Asia breeds. Furthermore, the supervised Admixture with prior information given to the three macro-regions Asia, Europe and Sub-Saharan Africa (colored bars in Figure 1B) reproduced the results of MDS (Figure 1A). These findings consistently highlighted a low genetic variability among Maghrebin breeds (Algeria, Morocco, and Tunisia), which share more ancestry with southern European (Iberian, Aegean, and Balkan) breeds than with sub-Saharan breeds. Two Maghreb breeds were clearly distinct from NA populations: the Tunisian NOTH sheep individuals were clustered with southern European breeds and the Algerian Sidaoun (SIDA) was similar to sub-Saharan breeds. Moreover, we observed a clear genetic differentiation of Libyan and Egyptian fat-tailed breeds (BARL, EGYB, and OSSI) from Maghrebin breeds which were rather closer to sheep breeds from the Middle East. The Tunisian Barbarine (BART), despite being close to the other thin-tailed breeds from the Maghreb, was found to be the one more “attracted” by the South-Eastern Mediterranean breeds. The ASD-NJ phylogenetic tree highlighted in Figure 2 the genetic relationship between local NA breeds and 300 fat-tailed sheep from Africa (Figure 2) shows that there is a separation according to the tail type, but with the Libyan and Tunisian Barbarine close to Maghrebin thin-tailed breeds and some Algerian Barbarine were located within these thin-tailed samples. The level of NA ancestry in some European fat-tailed sheep samples originating from Spain (ROMA), Italy (LATI, BASC), Greece (CHIO, KYMI, LESV), and Romania (KARO) is shown in Supplementary Figure S1. We found that the European fat-tailed breeds except the Aegean breeds were assigned to the North African sheep breed component, thus confirming the close relationship between the northern and southern shores of the Mediterranean Sea. As a consequence, the North African region is considered a crossroad of sheep genetic resources between Europe and Asia via continuous migration routes through the Mediterranean Sea.

### 3.2 Detection of geographic gradients

**Figure 3:**
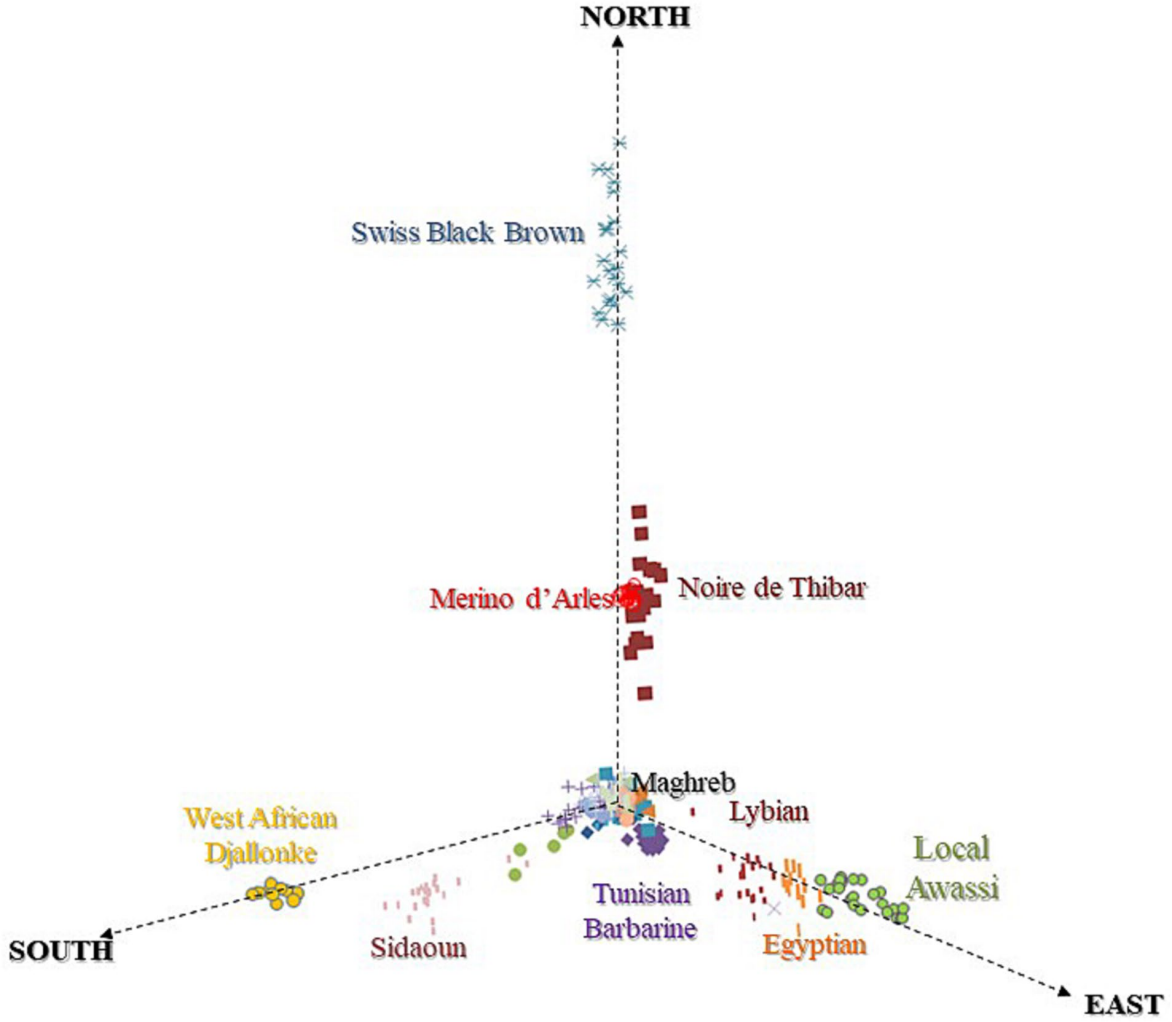
MDS plot of North African sheep breeds based on IBS distances revealing North-East-South geographic gradients.

The MDS plot (Figure 3) of NA sheep with breeds at the cross-border level suggested southward (Maghreb → Sahelian) geographic gradients that highlighted three main clines:

1. An immigration of MEAR and SWBB from the north, responsible for European ancestry of NOTH. The positioning of NOTH likely reflects the impact of European introgression and its historical origin as a crossbred between Maghrebin and MEAR.
2. A gene flow from northeast Africa, occupied by fat-tailed breeds (Egyptian, Libyan, and Local Awassi) with Asiatic ancestry.
3. A southward genetic cline toward the Sahel occupied by ancient hair sheep representing a sub-Saharan ancestry (SIDA and WAD). In the center of the plot, we found an overlapping cluster of the Maghrebin sheep breeds located at the crossing point between three directions.

In Supplementary Figure S2, the importance of west to east and vice versa geographic gradients is highlighted. Ordering the unsupervised admixture at *K* = 3 according to the westward component reveals a gradient of WAD to NOTH from south to north (Supplementary Figure S2A), whereas the largest westward components were observed in SIDA, Maghrebin DMAN, and some QFOT samples. In the bottom panel (Supplementary Figure S2B), a clear East–West gradient can be observed with Egyptian breeds being assigned the eastward component followed by Libyan Barbarine and the Tunisian Barbarine fat-tailed sheep breeds. Notably, BART had a lesser impact on genetic erosion and still conserved its genetic integrity in comparison with the Algerian Barbarine breed. The dispersal of fat-tailed breeds was indeed not complete, because Morocco only harbors thin-tailed breeds.

**Figure 4:**
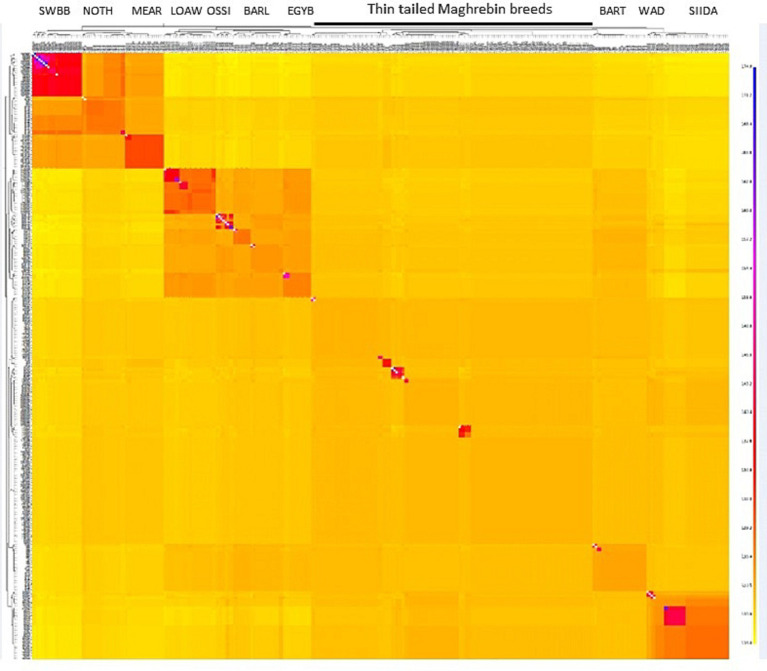
FineStructure clustering of NA sheep breeds. The color of each bin in the matrix indicates the number of “genomic chunks” copied from a donor (columns) to a recipient individual (rows).

To refine our perspective on genetic cline findings, the co-ancestry heatmap (Figure 4) represents the number of shared genomic ‘chunks’ between samples where darker/bluish colors indicate higher co-ancestry estimates, and the yellower colors the lower ones. The tree and chunk colors identify clusters based on haplotype sharing, confirming the clear genetic proximity between Maghrebin populations and highlighting a clustering of breeds located northward (Noire de Thibar-Merinos d’Arles and Swiss Black Brown), eastward (Libyan and Egyptian fat tailed), and southward (SIDA and WAD) on the North-East-South geographic gradient in Figure 3.

### 3.3 Footprints of selection in Maghrebin sheep breeds

#### 3.3.1 SNP loci under differential selection

The results of significant regions that were shared by at least three out of the six pairwise F_ST_-outlier contrasts of fat storage and coat pigmentation, respectively, are shown in Supplementary Tables S3, S4. Contrasting fat-tailed BART to five thin-tailed breeds revealed a total of 23 significant SNPs, most of which were identified in NOTH samples. The 200 kbp genomic regions upstream and downstream of the 23 significant markers are distributed along 10 chromosomes spanning 73 candidate genes. Remarkably, on chromosome 13, two loci rs427301675 (48,552,093 bp) and rs422598859 (48,935,908 bp) were significant in overall pairwise contrasts. These two loci are positioned in an interval (48231519- 49070447pb) of nine significant SNPs and span *BMP2*, *LOC101117953, LOC101118207*, and *LOC101110166* genes. The results of F_ST_ outlier loci contrasting NOTH breed to five white coat breeds revealed a total of 31 genomic regions spanning 206 genes on 16 chromosomes. At the chromosome level, the highest number of SNPs was observed on OAR 10. Only two regions are shared by all five pairwise contrasts. The first locus rs429070476 (69,867,326 bp) is located on OAR 6 and was identified as a region harboring *PDGFRA*, *LOC106990548*, and *LOC105613064*. The second marker rs193639663 (35,870,185 bp) is located on OAR10 and identified a region with 13 genes (*MICU2, LOC105611671, ZDHHC20, MRPL57, SKA3, TRNAE-UUC, SAP18, LOC101117678, LOC105611673, LATS2, XPO4, N6AMT2, and IFT88*). The results shown in Supplementary Tables S3, S4 show five common significant genomic regions harboring candidate genes under selection on OAR 1 (*UBE2Q1* and *ADAR*), OAR3 (*SOX10, PICK1*), OAR6 (*PDGFRA*), OAR15, (*PDGFD*) and OAR 20 (*MRPS18A* and *VEGFA*).

#### 3.3.2 European local ancestry in noire de Thibar breed

**Figure 5:**
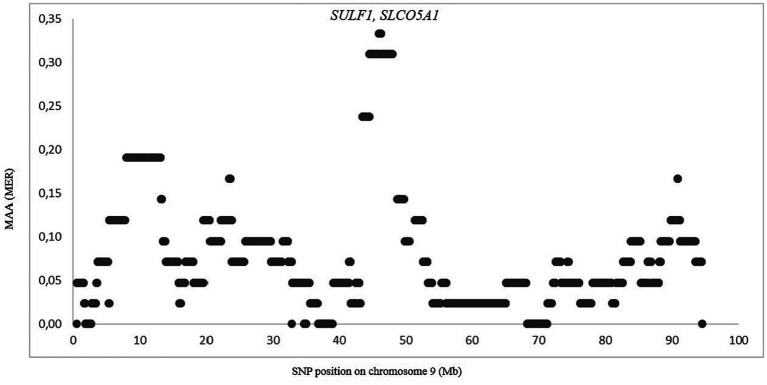
Zoom on chromosome 9 showing the merinos selection signature in position 45.9–46.3 Mb. Each dot represents a SNP position according to its MAA value.

The genomes of crossbred (admixed) individuals are a mosaic of ancestral haplotypes formed by recombination in each generation. The local sheep called ‘Noire de Thibar’ or ‘Black Thibar’(NOTH) presents a model example of a recently admixed sheep population, to create animals uniformly black to tolerate skin photosensitivity through a crossbreeding program between local white-coated QFOT and black French MEAR breeds. In 1970, a new gene pool was introduced into the breed using the SWBB breed in order to fix the black coat color, avoid consanguinity, and improve meat and wool quality. The LAMP-ANC analysis focused on the repartition of MAA proportions of the three parental breeds (QFOT, MEAR, and SWBB) in chromosomes of the admixed NOTH breed. The analysis of the global fraction of three parental breeds estimated 81% local Tunisian, 17% French Merinos, and 2% Swiss Black Brown (Table 1). The results pointed to an excess of QFOT locus-specific ancestry followed by MEAR and to a lower extent the SWBB. The LAMP results supported by two parental breeds with either MEAR or SWBB with QFOT as a common ancestor reveal a minimum of MAA of 0.07–0.33 and 0.21–0.43 for MEAR and SWBB, respectively (data not shown). The LAMP results identified putatively selected regions with the highest MAA levels (MAA = 0.33) genome-wide via MEAR ancestry in chromosome 9, including eight adjacent SNPs at position 45,961,627–46,302,319 bp which harbored two candidate genes, *SULF1* and *SLCO5A1* (Figure 5). Supplementary Table S5 shows the potential genomic regions under selection in NOTH samples issued from SWBB (MAA = 0.36–0.43). On OAR16, we noticed the highest locus-specific ancestry (MAA = 0.43) within the genomic region at position 65,952,055-66258912 bp harboring two uncharacterized genes *LOC105602639*, *LOC105602640* followed by the region (21579079–21,853,327 bp, MAA = 0.40) on OAR 21 (*SLC17A6, LOC106991834, ANO5* genes). Moreover, we noticed a moderate signal on OAR 16 (MAA = 0.38) spanning the region 67.4–67.8 Mb including two candidate genes *ICE1* and *ADAMTS16*. Remarkably, there was a partial overlapping of the previously detected signal MEAR local ancestry in the position 47,346,338-47971013 bp spanning 11 adjacent loci with MAA = 0.36 harboring *EYA1, LOC105607365* genes.

## 4 Discussion

### 4.1 Genetic relationships between North African and worldwide sheep breeds

Our current dataset contains the most comprehensive and largest collection of sheep breeds from NA currently available for global context population genetic studies. In this study, we provided a closer examination of admixture patterns, and genetic relationships among worldwide breeds, with a special focus on NA breeds. The global genetic structure reflects the geographic origin of most of the sheep breeds and reveals a genetic affinity of NA breeds to southern European/Mediterranean breeds rather than African breeds. These genetic patterns could be due to the substantial enrichment of the gene pool via maritime migrations throughout the Mediterranean Sea (49). This supports the importance of extensive historical exchanges during the history of mankind and livestock (4). Thus, the known historical route of migrations of sheep populations and gene flow exchange through the Mediterranean Sea did not displace or homogenize the genetic variation of North African sheep ancestors but rather enriched it. Indeed, (21) suggests direct contact between Balkan and some southern Italian breeds such as Barbaresca and Laticauda breeds which are known to have been influenced by NA fat-tailed Barbary sheep (50). Global-scale genetic diversity shows that Maghrebin sheep breeds overlap genetically with each other, reflecting global genetic homogenization and weak genetic structure among Maghreb sheep stocks (6, 25, 51). However, the differentiation of overall Maghrebin from Egyptian and Libyan sheep with affinity to Middle Eastern and Asian breeds may suggest two complementary hypotheses. According to the first hypothesis, the Tunisian and Algerian Barbarines are influenced by local thin-tailed breeds by genetic intermixing of fat- and thin-tailed breeds that appears to be a common feature in North African sheep, especially in the northwestern region (28, 52, 53). The second hypothesis suggests that the geographic isolation by adaptation to different eco-climates as well as different cultural practices have differentiated native sheep populations (22). At the local geographic scale, the genetic structure of NA breeds mirrors a differential mixture of genetic backgrounds relative to the origin of individual breeds showing historical directional migrations. Thus, the northern genetic cline is shaped by the presence of NOTH attracted by two European breeds (MEAR and SWBB), confirming the influence of introgression of European stocks MEAR (black wool) and the Swiss Black Brown Mountain. Previous studies support the genetic differentiation of the NOTH breed between local Tunisian breeds (7, 28) and other Maghrebin sheep populations (25). In addition, the southward trajectory has created a continuous cline of Maghreb via SIDA and the sub-Sahelian primitive WAD sheep. Indeed, the Sidaoun subpopulation “Terguia-Sidaou” sheep derived its name from the Touareg tribes in the Sahara in the Libyan Fezzan desert, Niger and the Hoggar-Tassili area in southern Algeria. The origin of the breed is believed to be in Sudan and it may be related to the Sudanese Peul breed (54). Furthermore, archeological and anthropological evidence indicates two separate introductions of fat-tail sheep into the African continent, along the Mediterranean Sea coastline and via the Horn of Africa after crossing the strait of Babel- Mandeb, respectively (22). Furthermore, the Arabian invasion in the 7th century AD may very well have exerted a significant impact on sheep husbandry in North Africa and the Iberian peninsula (11), although this has not yet been detailed on the basis of molecular data.

### 4.2 Identification of potential genes under selection

The obtained genes were shared in at least three comparisons between breeds/groups of breeds, providing evidence that they are not artifacts but potential genomic regions affected by selection (55). Genes that underlined differences between fat-tailed Barbarine and thin-tailed breeds are associated with fat distribution (*BMP2, PDGFD, VEGFA, TBX15*, and *WARS2*) and horn morphology and polledness [*RXFP2*, relaxin/insulin like family peptide receptor 2, (17)]. In fact, a comparison of Afec-Assaf sheep and its parental Awassi breed revealed variation in or near *PDGFD, VEGFA*, and *RXFP2* (36). The *BMP2* and *PDGFD* (platelet-derived growth factor D) genes are considered plausible candidate genes for the sheep fat-tail phenotype (10, 56, 57–59, 60–62). The *VEGFA* (vascular endothelial growth factor A) gene is in endothelial cells involved in angiogenesis by targeting lipids to peripheral tissues and may thus influence the expansion and loss of adipose tissue (63) and fat distribution in humans (64). Increased *VEGFA* expression was considered to contribute to genetic adaptation to hypoxia in high-altitude regions (65, 66). The *TBX15* and *WARS2* genes have been associated with fat distribution at the waist and hip in humans (64), whereas the *TBX15* gene is also associated with adipocyte differentiation and mitochondrial respiration (67), indicating that these genes also play a role in fat deposition in sheep tails. On OAR1, we identified *UBE2Q1* and *ADAR* genes, which were also detected in Russian Romanov sheep. The *UBE2Q1 gene* is implicated in embryo implantation, fertilization, and prolactin secretion and the *ADAR* gene is related to osteoblast differentiation (68). On OAR 9, the *PLAG1* (pleomorphic adenoma gene 1) seems to be more related to early developmental events (69). It is primarily expressed during embryonic development and has been reported to have an effect on the growth performance in mice, cattle, pigs, and sheep (70). This study identified several candidate genes under strong selection associated with melanocyte activity and pigmentation (*SOX10, PICK1, PDGFRA, and MC1R*) that were previously detected as selection signatures related to pigmentation traits using GBS data (13). Indeed, the *PDGFRA* gene interacts in the functional pathway of coat color in different mammals and plays an important role in determining white coat color in Iranian goats (71). Moreover, we detected on OAR19 the *MTIF* (micropthalmia transcription factor) gene. The MITF protein regulates melanocyte development and is responsible for pigment cell-specific transcription of the melanogenesis enzyme genes. It has been involved in melanogenesis and melanoma angiogenesis in different mammalians (72).

### 4.3 Signature of “marinization” through analysis of local genome ancestry

The local ancestry approach is particularly suitable for detecting regions subject to selection in a crossbred population in which traits of one of the ancestral populations have been selected. This approach has been successfully used to detect recent selection in admixed cattle populations (73–75) and in Merino sheep (76). Our analysis of NOTH confirmed its composite origin with about 17% MEAR introgression, which likely was introduced at the beginning of the last century to improve the wool fiber quality, and a more minor SWBB introgression in order to restore its black color (77, 78). We found MEAR and SWBB-derived selection signals on OAR9 and OAR16, respectively. Interestingly, the selection signal in region 45.9–46.3 Mb on OAR 9 spanned the *SULF1* gene, which is known as the candidate gene involved in primary wool follicle induction and skin development (79, 80). The same signal was also detected via SWBB local ancestry, which may be explained by the origin of SWBB as a cross of Flemish Landrace sheep and Spanish Black Merinos at the beginning of the 19th century (25). The *SULF1* gene encodes an enzyme of the sulfatase class able to alter the sulfation patterns of proteoglycans and consequently the binding site of many growth factors such as WNT, BMP, and FGF (81). These are known to play major roles during embryogenesis and during hair follicle development. Thus, the *SULF1* haplotype may be considered as a signal of “merinization” that is selected to improve the wool fiber quality. The *ADAMTS16* gene on OAR16 is associated with high altitude adaptation in Chinese pig breeds (82). The *ICE1* gene, also on OAR16, has been identified as a key gene in the cold acclimation pathway in live oak trees (83) and as a regulator of cold-induced transcriptome and freezing tolerance in Arabidopsis (84). Therefore, the adaptation to high altitude and low temperature may be important for local adaptation to the sub-humid climate in the center of NOTH breeding in the north of Tunisia.

## 5 Conclusion

Our findings improve our understanding of the composition and origins of North African sheep and bridge the gap in previous global-scale genomic studies between the north and south shores of the Mediterranean Sea. Although the adaptive traits require further validation, our results may already be used in genomic-based breeding programs to improve resilience traits in North African sheep. In summary, our study provides important clues for devising better strategies for sustainable conservation and management of genetic resources while supporting the local rural economy.

## Data availability statement

The original contributions presented in the study are publicly available. This data can be found at: DOI: 10.6084/m9.figshare.24588240.

## Ethics statement

Ethical approval was not required for the study involving animals in accordance with the local legislation and institutional requirements because animal samples were obtained in compliance with local/national laws in force at the time of sampling. Blood sampling was carried out by trained veterinarians or under veterinarian supervision within the frame of vaccination campaigns, hence no permission from the animal research ethics committee was necessary.

## Author contributions

IB: Data curation, Formal analysis, Software, Writing – original draft. SB-R: Conceptualization, Resources, Supervision, Writing – review & editing. SM: Investigation, Software, Writing – review & editing. JL: Data curation, Writing – review & editing, Investigation. AS: Data curation, Formal analysis, Writing – review & editing. BB: Data curation, Writing – review & editing. EC: Conceptualization, Data curation, Formal analysis, Project administration, Supervision, Writing – review & editing.

## Footnotes

^1^ https://www.cog-genomics.org/plink/

^2^ https://www.ncbi.nlm.nih.gov/genome/gdv/browser/genome/?id=GCF_016772045.1
