# Supplementary material for: Refining the genomic profiles of North African sheep breeds through meta-analysis of worldwide genomic SNP data
Source: Front Vet Sci. 2024 Feb 29;11:1339321. doi: 10.3389/fvets.2024.1339321 (PMC10938946; doi:10.3389/fvets.2024.1339321)
Supplement: Supplementary file 2 [file Table_2.docx]

**Supplementary Table S2.** Sheep breeds used in pairwise comparisons using Bayescan “F_ST_-outlier” approach

| **Trait of interest** | **Tunisian breed** | **Contrasted breed^1^** | **Origin** |
| --- | --- | --- | --- |
| Tail fatness | BART | NOTH  QFOT  NOTH + QFOT  BERG  TIBT  WAD | Tunisia  Tunisia  Tunisia  Europe  Asia  West Africa |
| Coat pigmentation | NOTH | BART  QFOT  BART + QFO  ODJA+ODMA  BERG  CHANG | Tunisia  Tunisia  Tunisia  North Africa  Europe  Asia |

^1^ we used six pairwise comparisons between
