# Supplementary material for: Refining the genomic profiles of North African sheep breeds through meta-analysis of worldwide genomic SNP data
Source: Front Vet Sci. 2024 Feb 29;11:1339321. doi: 10.3389/fvets.2024.1339321 (PMC10938946; doi:10.3389/fvets.2024.1339321)
Supplement: Supplementary file 3 [file Table_3.docx]

**Supplementary Table S3.** Results of the F_ST_-outlier analysis performed contrasting Tunisian Barbarine breed with thin-tailed sheep breeds (QFO, NOTH, BERG, TIBT and WAD).

|  | | Thin-tailed breeds | | | | | | Position (bp) | Genomic regions | Genes |
| --- | --- | --- | --- | --- | --- | --- | --- | --- | --- | --- |
| OAR | SNP | QFO | NDT | QFO+NDT | BERG | TIBT | WAD |  |  |  |
| 1 | rs415409374 |  | x | x |  | x |  | 103455454 | 103255454-103655454 | *UBE2Q1, LOC106990860, CHRNB2, ADAR, LOC105608947, KCNN3, PMVK, PBXIP1, LOC101104627, PYGO2, SHC1* |
| 2 | rs410510988 |  | x | x | x |  |  | 104273138 | 104073138-104473138 | *FAM167A, BLK, GATA4, NEIL2, FDFT1, CTSB* |
| 2 | rs399829205 | x |  | x | x |  |  | 105994000 | 105794000-106194000 | *LOC105608584, HAND2, LOC105608588* |
| 2 | rs398461786 |  | x | x | x |  |  | 107043868 | 106843868-107243868 | *GALNTL6* |
| 3 | rs398298116 |  | x | x | x |  |  | 213562255 | 213362255-213762255 | *LOC101102223, GCAT, GALR3, ANKRD54, EIF3L, MICALL1, C3H22orf23, POLR2F, SOX10, LOC105609490, LOC105614868, PICK1, SLC16A8, BAIAP2L2, PLA2G6, MAFF, TMEM184B* |
| 6 | rs426192522 |  | x | x | x | x |  | 35970906 | 35770906-36170906 | *FAM13A, HERC3, NAP1L5, PYURF, PIGY,HERC5* |
| 6 | rs429070476 |  | x | x |  | x |  |  | 69667326-70067326 | *PDGFRA, LOC106990548, LOC105613064* |
| 9 | rs405656050 |  | x | x | x |  |  | 36057235 | 35857235-36257235 | *LYN, RPS20, MOS, PLAG1, CHCHD7, SDR16C5, LOC101116323* |
| 9 | rs427884086 |  | x | x | X |  |  | 36179509 | 35979509-36379509 | *RPS20, MOS, PLAG1, CHCHD7, SDR16C5, LOC101116323, PENK* |
| 10 | rs410444662 |  |  |  | X | x | x | 29588481 | 29388481-29788481 | *LOC106991357, LOC101110773, RXFP2, LOC106991379* |
| 10 | rs404084643 |  | x | x | X |  |  | 30648329 | 30448329-30848329 | *LOC101112330, LOC101112071, TRNAW-CCA, KATNAL1* |
| 12 | rs426920184 |  | x | x |  |  | x | 21053195 | 20853195-21253195 | _ |
| 13 | rs403860093 | x | x | x | X |  |  | 48231519 | 48031519-48431519 | *BMP2* |
| 13 | rs409368932 |  | x | x | X |  |  | 48294783 | 48094783-48494783 | *BMP2* |
| 13 | rs404995502 | x | x | x |  |  |  | 48420340 | 48220340-48620340 | *BMP2* |
| 13 | rs427301675 | x | x | x | x | x | x | 48552093 | 48352093-48752093 | *BMP2* |
| 13 | rs407110393 | x | x | x |  |  |  | 48696401 | 48496401-48896401 | _ |
| 13 | rs417470080 | x | x | x |  |  |  | 48761402 | 48561402-48961402 | *LOC101117953* |
| 13 | rs399507852 | x | x | x | X |  | x | 48897111 | 48697111-49097111 | *LOC101117953, LOC101118207* |
| 13 | rs422598859 | x | x | x | X | x | x | 48935908 | 48735908-49135908 | *LOC101117953, LOC101118207, LOC101110166* |
| 13 | rs419628794 | x | x | x | X | x |  | 49070447 |  | *LOC101117953, LOC101118207, LOC101110166* |
| 15 | rs400618975 | x | x | x |  |  |  | 3709662 | 3509662-3909662 | *PDGFD* |
| 20 | rs423742731 | x | x | x |  | x |  | 17382988 | 17182988-17582988 | *MRPS18A, LOC105603710, VEGFA, C20H6orf223, LOC106990495* |
