# Supplementary material for: Refining the genomic profiles of North African sheep breeds through meta-analysis of worldwide genomic SNP data
Source: Front Vet Sci. 2024 Feb 29;11:1339321. doi: 10.3389/fvets.2024.1339321 (PMC10938946; doi:10.3389/fvets.2024.1339321)
Supplement: Supplementary file 4 [file Table_4.docx]

**Supplementary Table S4**. Results of the F_ST_-outlier analysis performed contrasting Noire de Thibar breed with six white coat sheep breeds (BART, QFO, ODJ, BERG and CHANG)

|  | | White coat breeds | | | | | | Position | Genomic regions | Genes |
| --- | --- | --- | --- | --- | --- | --- | --- | --- | --- | --- |
| OAR | SNP | BART | QFO | BART+QFO | ODMA+ODJA | BERG | CHANG |  |  |  |
| 1 | rs429356877 | x |  | x |  |  | x | 95706894 | 95506894-95906894 | *TBX15, WARS2, HAO2* |
| 1 | rs415409374 | x |  | x |  | x |  | 103455454 | 103255454-103655454 | *UBE2Q1, LOC106990860, CHRNB2, ADAR, LOC105608947, KCNN3, PMVK, PBXIP1, LOC101104627, PYGO2, SHC1* |
| 2 | rs402017591 | x | x | x |  |  |  | 194581057 | 194381057-194781057 | *-* |
| 2 | rs403837881 |  | x | x |  | x |  | 196560207 | 196360207-196760207 | *-* |
| 3 | rs406760597 | x | x | x |  |  |  | 209593617 | 209393617-209793617 | *FGF6, FGF23, TIGAR, CCND2, LOC105608579, LOC105608577* |
| 3 | rs398298116 | x |  | x | x |  | x | 213562255 | 213362255-213762255 | *LOC101102223, GCAT, GALR3, ANKRD54, EIF3L, MICALL1, C3H22orf23, POLR2F, SOX10, LOC105609490, LOC105614868, PICK1, SLC16A8, BAIAP2L2, PLA2G6, MAAF, TMEM184B* |
| 4 | rs401097721 | x |  | x | x |  |  | 48649775 | 48449775-48849775 | *COG5, GPR22, LOC106991126, DUS4L, BCAP29, SLC26A4, LOC10511148, CBLL1, SLC26A3* |
| 4 | rs413699682 | x | x | x |  |  |  | 105095036 | 104895036-105295036 | *CLEC5A, LOC101113144, MGAM, MGAM2, LOC101114187, LOC101111274, LOC101111538, LOC101111795, LOC101112049, LOC101114438, LOC101114950, LOC105613885, LOC101115704, LOC101116724, LOC101116984* |
| 5 | rs430011729 | x | x |  | x |  |  | 24839140 | 24639140-25039140 | *TEX43, PHAX, ALDH7A1, GRAMD3, LOC105615237, LOC105608683* |
| 6 | rs424702571 | x |  | x |  |  | x | 51603405 | 51403405-51803405 | *LOC101107868* |
| 6 | rs417737324 | x | x | x |  |  |  | 69816517 | 69616517-70016517 | *PDGFRA, LOC106990548, LOC105613064* |
| 6 | rs429070476 | x | x | x | x | x |  | 69867326 | 69667326-70067326 | *PDGFRA, LOC106990548, LOC105613064* |
| 7 | rs429758116 | x | x | x |  |  |  | 21428030 | 21228030-21628030 | *HOMEZ, RNF212B, LOC101121938,SLC7A8, CEBPE, C7H14orf119, ACIN1, LOC105608568, CDH24, PSMB11, LOC101123213, PSMB5, C7H14orf93, AJUBA, HAUS4, TRNAR-ACG, PRMT5, RBM23, REM2, LRP10* |
| 7 | rs399281941 | x |  | x | x |  |  | 79996255 | 79796255-80196255 | *SIPA1L1, LOC105615765* |
| 8 | rs430521717 | x |  | x | x |  |  | 31710719 | 31510719-31910719 | *PREP, LOC105611049, POPDC3* |
| 10 | rs421468589 | x |  | x |  | x | x | 22940958 | 22740958-23140958 | *LOC105608780, LOC101122286, COG6, LHFP* |
| 10 | rs414336311 | x |  | x |  | x |  | 30591945 | 30391945-30791945 | *TRNAN-GUU, USPL1, LOC101112330, LOC101112071, TRNAW-CCA, KATNAL1* |
| 10 | rs193639663 | x | x | x | x | x |  | 35870185 | 35670185-36070185 | *MICU2, LOC105611671, ZDHHC20, MRPL57,SKA3, TRNAE-UUC, SAP18, LOC101117678, LOC105611673, LATS2, XPO4, N6AMT2, IFT88* |
| 10 | rs414637089 | x | x | x |  | x |  | 35977709 | 35777709-36177709 | *SKA3, TRNAE-UUC, SAP18, LOC101117678, LOC105611673, LATS2, XPO4, N6AMT2, IFT88, CRYL1* |
| 10 | rs419789292 | x | x | x | x |  |  | 36196692 | 35996692-36396692 | *N6AMT2, IFT88, CRYL1, LOC101118964, LOC105611675, GJB6, GJB2, GJA3, ZMYM2* |
| 11 | rs410999851 | x | x | x |  |  |  | 21094375 | 20894375-21294375 | *SLC6A4, BLMH, TMIGD1, CPD, LOC106991395, GOSR1, TUSC5, BHLHA9,ABR* |
| 14 | rs418443666 | x | x | x |  |  |  | 14160697 | 13960697-14360697 | *SPG7, RPL13, CPNE7, DPEP1, CHMP1A, SPATA33, CDK10, SPATA2L, ZNF276, VPS9D1, FANCA, SPIRE2, TCF25, MC1R, DEF8, CENPBD1, LOC101113264, DBNDD1, GAS8, LOC101109035, SHCBP1* |
| 14 | rs398241949 |  | x | x | x |  |  | 14274099 | 14074099-14474099 | *ZNF276, FANCA, SPIRE2, TCF25, MC1R, DEF8, CENPBD1, LOC101113264, DBNDD1, GAS8, LOC101109035, SHCBP1, VPS35* |
| 15 | rs400618975 | x |  | x |  | x |  | 3709662 | 3509662-3909662 | *PDGFD* |
| 15 | rs421429495 | x |  | x | x |  |  | 39088847 | 38888847-39288847 | *ARNTL, TRNAE-CUC, RASSF10, TEAD1* |
| 15 | rs411208945 | x |  | x |  |  | x | 74220070 | 74020070-74420070 | *CRY2, MAPK8IP1, C15H11orf94,PEX16, GYLTL1B, PHF21A, CREB3L1* |
| 16 | rs410165873 | x | x | x |  |  |  | 65806994 | 65606994-66006994 | *ADCY2* |
| 16 | rs399773623 | x |  | x |  | x |  | 61441351 | 61241351-61641351 | *-* |
| 19 | rs419333175 | x |  | x |  | x |  | 31614145 | 31414145-31814145 | *LOC105607729, MITF, LOC105603449* |
| 20 | rs423742731 | x |  | x | x | x |  | 17382988 | 17182988-17582988 | *MRPS18A, LOC105603710, VEGFA, C20H6orf223, LOC106990495* |
| 24 | rs423068203 | x | x | x | x |  |  | 33931727 | 33731727-34131727 | *POM121C, LOC105604773, HIP1, LOC105604774, CCL26, CCL24, TRNAE-CUC, RHBDD2, POR, LOC1069911916, TMEM120A, STYXL1* |
