# Supplementary material for: Refining the genomic profiles of North African sheep breeds through meta-analysis of worldwide genomic SNP data
Source: Front Vet Sci. 2024 Feb 29;11:1339321. doi: 10.3389/fvets.2024.1339321 (PMC10938946; doi:10.3389/fvets.2024.1339321)
Supplement: Supplementary file 5 [file Table_5.docx]

**Supplementary Table S5.** Details of potential genomic regions under selection in NOTH samples issue from SWBB (MAA=0.36-0.43).

| Chromosome | start position | End position | SNP ID start | SNP ID end | SNPs | Top MAA_SWBB | | Genes in the region |
| --- | --- | --- | --- | --- | --- | --- | --- | --- |
| 9 | 6872283 | 9272742 | rs409789319 | rs412865040 | 32 | | 0.40 | *LOC101103735* |
|  | 44538785 | 47971013 | rs425012839 | rs408230212 | 60 | | 0.36 | *LOC101102990, PREX2, LOC105616041, C9H8orf34, SULF1, SLCO5A1, TRNAG-CCC, PRDM14, LOC101103989, NCOA2, TRAM1, LACTB2, XKR9, LOC10111527, LOC106991338,* ***EYA1,*** *LOC105607365* |
|  | 65952055 | 66258912 | rs409900768 | rs398454538 | 9 | | 0.43 | *LOC105602639, LOC105602640* |
| 16 | 67418421 | 67888612 | rs420939182 | rs408347910 | 8 | | 0.38 | *ICE1, LOC105602647, ADAMTS16* |
