# Supplementary material for: Refining the genomic profiles of North African sheep breeds through meta-analysis of worldwide genomic SNP data
Source: Front Vet Sci. 2024 Feb 29;11:1339321. doi: 10.3389/fvets.2024.1339321 (PMC10938946; doi:10.3389/fvets.2024.1339321)
Supplement: Supplementary file 6 [file Image_1.pdf]

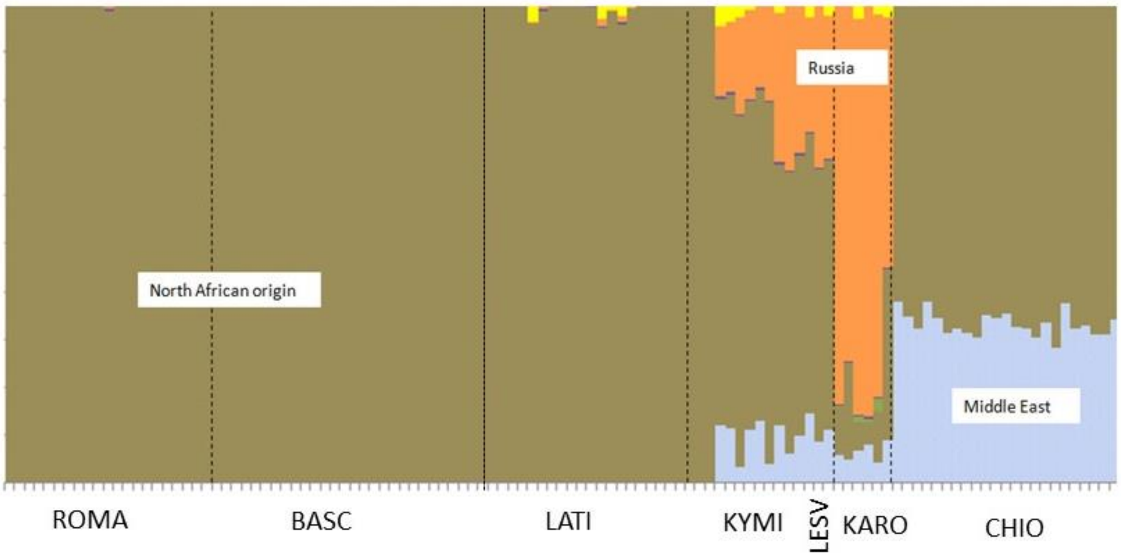

Supplementary Figure 1. Admixture supervised (K=3) of European fat-tailed sheep breeds originated from Spain (ROMA), Italy (LATI, BASC), Greece (CHIO, KYMI, LESV) and Romania (KARO) with prior information given to breeds from North Africa, Middle East and Russian ancestry.
