# Supplementary material for: Refining the genomic profiles of North African sheep breeds through meta-analysis of worldwide genomic SNP data
Source: Front Vet Sci. 2024 Feb 29;11:1339321. doi: 10.3389/fvets.2024.1339321 (PMC10938946; doi:10.3389/fvets.2024.1339321)
Supplement: Supplementary file 7 [file Image_2.pdf]

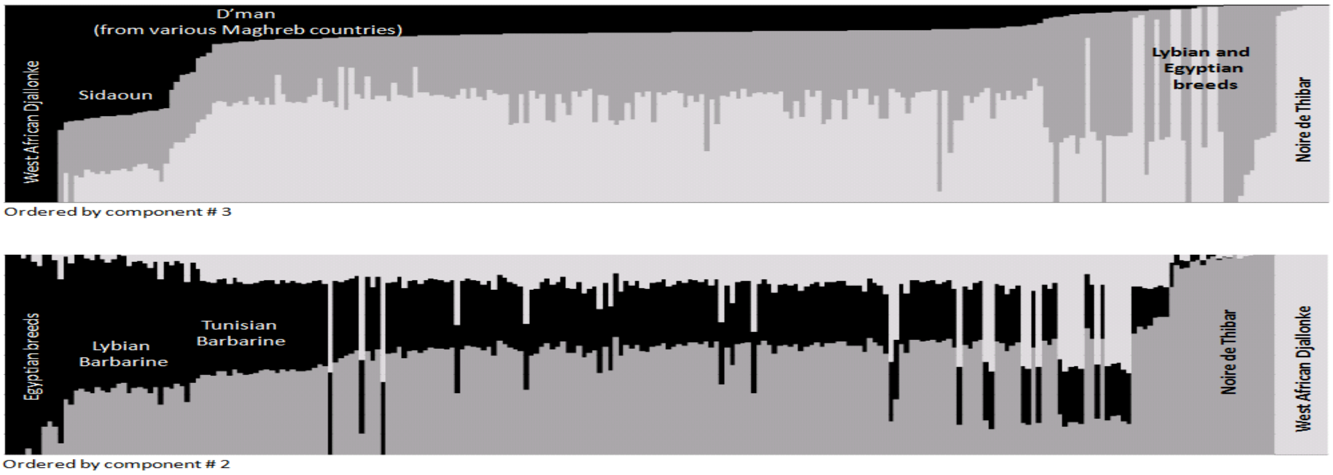

Supplementary Figure 2. Admixture unsupervised k=3 showing west-east gradient in northern African sheep breeds. (A) Top panel ordered by component 3 representing the southward component assigned to WAD. (B) Bottom panel ordered by component 2 representing the eastward component corresponding to fat tailed Egyptian breeds
